# Supplementary material for: The Combination of Conventional QTL Analysis, Bulked-Segregant Analysis, and RNA-Sequencing Provide New Genetic Insights into Maize Mesocotyl Elongation under Multiple Deep-Seeding Environments
Source: Int J Mol Sci. 2022 Apr 11;23(8):4223. doi: 10.3390/ijms23084223 (PMC9032596; doi:10.3390/ijms23084223)
Supplement: Supplementary file 1 [file ijms-23-04223-s001.zip › ijms-1642070-SI.pdf]

**Table S1.** Phenotype, broad-sense heritability, and genotype × environment interaction heritability of mesocotyl length in $F_{2:3}$ families under 3 cm, 15 cm, and 20 cm sowing depth environments.

| Sowing depth environment | Mean | Range | CV (%) | Skewness | Kurtosis | $H_B^2$ | $H_{GE}^2$ |
|---|---|---|---|---|---|---|---|
| 3 cm | 3.74±0.61 | 0.72~5.51 | 16.31 | 0.112 | 0.110 | 90.96 | 8.98 |
| 15 cm | 10.95±2.57 | 3.30~15.75 | 23.47 | 0.450 | 0.301 | | |
| 20 cm | 13.09±5.88 | 4.00~19.34 | 44.92 | 0.982 | 0.337 | | |

$H_B^2$, broad-sense heritability; $H_{GE}^2$, genotype × environment interaction heritability; CV, coefficient of variation.

**Table S2.** Quality control of sequencing data for female parent W64A, male parent K12, and bulked LM-pool and SM-pool samples under 20 cm sowing environment, respectively.

| Bulk | Clean reads | Data generated | Q30 (%) | GC (%) | Genome coverage (10×) | Averagedepth (×) | Alignment efficiency (%) |
|---|---|---|---|---|---|---|---|
| W64A | 217,228,830 | 65,168,649,000 | 93.32 | 46.46 | 76.30 | 34.02 | 99.59 |
| K12 | 198,744,069 | 59,623,220,700 | 93.10 | 45.81 | 76.78 | 30.85 | 99.48 |
| LM-pool | 282,373,223 | 84,711,966,900 | 93.66 | 45.98 | 86.08 | 41.10 | 99.58 |
| SM-pool | 342,998,856 | 102,899,656,800 | 93.20 | 46.02 | 87.39 | 50.10 | 99.13 |

**Table S3.** Annotation of variation SNPs information among W64A, K12, LM-pool, and SM-pool samples, respectively.

| Variation SNP information | Total | W64A | K12 | LM-pool | SM-pool |
|---|---|---|---|---|---|
| Downstream | 2,224,362 | 1,035,464 | 1,069,307 | 1,528,150 | 1,549,083 |
| Exon | 15,825 | 6,950 | 8,116 | 11,189 | 10,970 |
| Intergenic | 16,271,598 | 7,629,206 | 7,621,337 | 10,505,125 | 10,668,917 |
| Intron | 637,413 | 314,325 | 314,055 | 448,811 | 451,889 |
| Non_Synonymous_Coding | 244,472 | 119,191 | 118,942 | 174,520 | 176,522 |
| Splice_Site_Acceptor | 2,495 | 1,200 | 1,250 | 1,720 | 1,725 |
| Splice_Site_Donor | 2,218 | 1,059 | 1,069 | 1,569 | 1,585 |
| Start_Gained | 9,740 | 4,667 | 4,784 | 6,854 | 6,866 |
| Start_Lost | 1,042 | 547 | 525 | 788 | 773 |
| Stop_Gained | 6,284 | 2,743 | 2,825 | 4,060 | 4,079 |
| Stop_Lost | 1,181 | 629 | 622 | 876 | 886 |
| Synonymous_Coding | 223,641 | 109,906 | 110,909 | 165,227 | 166,827 |
| Synonymous_Stop | 436 | 230 | 240 | 311 | 314 |
| Unknown | 17,008 | 7,884 | 7,938 | 11,897 | 11,806 |
| Upstream | 3,195,437 | 1,474,433 | 1,555,230 | 2,213,902 | 2,241,915 |
| Utr_3_Prime | 229,368 | 110,127 | 116,900 | 168,725 | 170,172 |
| Utr_5_Prime | 190,512 | 92,257 | 96,055 | 139,171 | 140,432 |

**Table S4.** Annotation of variation InDels information among W64A, K12, LM-pool, and SM-pool samples, respectively

| Variation InDel information | Total | W64A | K12 | LM-pool | SM-pool |
|---|---|---|---|---|---|
| Codon_Change_Plus_Codon_Deletion | 4,764 | 4,555 | 4,256 | 4,687 | 4,726 |
| Codon_Change_Plus_Codon_Insertion | 2,580 | 2,494 | 2,340 | 2,539 | 2,562 |
| Codon_Deletion | 2,602 | 2,487 | 2,308 | 2,554 | 2,581 |
| Codon_Insertion | 4,378 | 4,204 | 3,977 | 4,331 | 4,358 |
| Downstream | 442,405 | 404,001 | 406,995 | 435,403 | 435,597 |
| Exon | 1,635 | 1,473 | 1,497 | 1,609 | 1,608 |
| Exon_Deleted | 13 | 13 | 11 | 13 | 13 |
| Frame_Shift | 18,223 | 17,178 | 16,857 | 18,007 | 18,060 |
| Gene_Fusion | 5 | 5 | 5 | 5 | 5 |
| Gene_Fusion_Reverese | 23 | 20 | 21 | 22 | 23 |
| Intergenic | 1,277,771 | 1,137,951 | 1,150,246 | 1,252,836 | 1,249,391 |
| Intron | 140,483 | 131,850 | 132,674 | 139,189 | 139,145 |
| Splice_Site_Acceptor | 959 | 894 | 906 | 953 | 946 |
| Splice_Site_Donor | 1,143 | 1,080 | 1,075 | 1,130 | 1,131 |
| Start_Lost | 110 | 105 | 104 | 109 | 110 |
| Stop_Gained | 229 | 210 | 207 | 225 | 227 |
| Stop_Lost | 81 | 76 | 79 | 80 | 81 |
| Unknown | 30,143 | 28,601 | 28,366 | 29,906 | 29,943 |
| Upstream | 689,777 | 628,463 | 632,705 | 678,423 | 678,950 |
| Utr_3_Prime | 50,622 | 47,914 | 48,119 | 50,181 | 50,199 |
| Utr_5_Deleted | 1 | 1 | 0 | 1 | 1 |
| Utr_5_Prime | 42,814 | 40,612 | 40,672 | 42,508 | 42,519 |

**Table S5.** Summary of quality preprocessing of RNA sequencing (RNA-Seq) data.

| Sample | Raw Date (bp) | Clean Date (bp) | Clean Date Rate (%) | Total Reads | Q20 (%) | Q30 (%) | GC (%) | Mapped Reads | Mapping Rate (%) |
|---|---|---|---|---|---|---|---|---|---|
| W64A(3 cm) | 7,010,755,947 | 6,941,989,700 | 100.44 | 47,020,862 | 98.23 | 94.76 | 53.91 | 42,455,639 | 90.43 |
| W64A(20 cm) | 8,190,515,323 | 8,192,650,400 | 99.56 | 47,635,087 | 98.23 | 94.77 | 54.72 | 42,527,764 | 89.33 |
| K12(3 cm) | 9,280,368,041 | 7,852,391,550 | 84.53 | 55,602,589 | 97.7 | 93.45 | 53.63 | 48,326,857 | 86.81 |
| K12(20 cm) | 9,114,478,837 | 6,954,957,300 | 76.32 | 52,798,507 | 98.14 | 94.52 | 53.29 | 47,129,827 | 89.27 |
| Mean | 8,399,029,537 | 7,485,497,238 | 90.21 | 50,764,261 | 98.08 | 94.38 | 53.89 | 45,110,022 | 88.96 |

**Table S6.** Go annotation of 24 core conserved differentially expressed genes (DEGs) in all four comparisons by RNA-sequencing (RNA-Seq).

| Gene ID | GO_annotation |
|---|---|
| Zm00001d039958 | Molecular Function: N-acyltransferase activity (GO:0016410);; |
| MSTRG.44705 | -- |
| Zm00001d016500 | -- |
| MSTRG.38120 | -- |
| Zm00001d002005 | Molecular Function: DNA binding (GO:0003677);; Cellular Component: nucleus (GO:0005634);; Biological Process: multicellular organism development (GO:0007275);; Molecular Function: metal ion binding (GO:0046872);; |
| Zm00001d016271 | Molecular Function: nucleotide binding (GO:0000166);; Molecular Function: RNA binding (GO:0003723);; Cellular Component: spliceosomal complex (GO:0005681);; Biological Process: mRNA processing (GO:0006397);; Biological Process: RNA splicing (GO:0008380);; Cellular Component: nuclear speck (GO:0016607);; |
| Zm00001d020206 | Biological Process: fatty acid biosynthetic process (GO:0006633);; Cellular Component: integral component of membrane (GO:0016021);; Molecular Function: 3-oxo-arachidoyl-CoA synthase activity (GO:0102336);; Molecular Function: 3-oxo-cerotoyl-CoA synthase activity (GO:0102337);; Molecular Function: 3-oxo-lignoceronyl-CoA synthase activity (GO:0102338);; |
| MSTRG.36969 | -- |
| Zm00001d048874 | Molecular Function: magnesium ion binding (GO:0000287);; Biological Process: defense response (GO:0006952);; Biological Process: metabolic process (GO:0008152);; Molecular Function: ent-copalyl diphosphate synthase activity (GO:0009905);; Molecular Function: terpene synthase activity (GO:0010333);; |
| Zm00001d023604 | -- |
| Zm00001d048631 | Molecular Function: protein serine/threonine kinase activity (GO:0004674);; Molecular Function: calcium ion binding (GO:0005509);; Molecular Function: ATP binding (GO:0005524);; Biological Process: cell surface receptor signaling pathway (GO:0007166);; Cellular Component: integral component of membrane (GO:0016021);; Molecular Function: polysaccharide binding (GO:0030247);; |
| MSTRG.21330 | -- |
| MSTRG.47158 | Cellular Component: integral component of plasma membrane (GO:0005887);; Biological Process: carbohydrate transport (GO:0008643);; Cellular Component: integral component of membrane (GO:0016021);; Molecular Function: sugar transmembrane transporter activity (GO:0051119);; |
| Zm00001d031738 | -- |
| Zm00001d049020 | Molecular Function: O-methyltransferase activity (GO:0008171);; |
| Zm00001d035172 | Molecular Function: nucleotide binding (GO:0000166);; Molecular Function: ATP binding (GO:0005524);; Cellular Component: plasma membrane (GO:0005886);; Biological Process: signal transduction (GO:0007165);; Biological Process: plant-type hypersensitive response (GO:0009626);; Cellular Component: endomembrane system (GO:0012505);; Cellular Component: extrinsic component of plasma membrane (GO:0019897);; Molecular Function: ADP binding (GO:0043531);; |
| Zm00001d003648 | Molecular Function: histone acetyltransferase activity (GO:0004402);; Cellular Component: nucleus (GO:0005634);; Biological Process: transcription, DNA-templated (GO:0006351);; Biological Process: regulation of transcription, DNA-templated (GO:0006355);; Molecular Function: zinc ion binding (GO:0008270);; Cellular Component: plasmodesma (GO:0009506);; Molecular Function: double-stranded methylated DNA binding (GO:0010385);; Molecular Function: histone binding (GO:0042393);; Biological Process: histone H3-K18 acetylation (GO:0043971);; Biological Process: histone H3-K23 acetylation (GO:0043972);; Biological Process: regulation of DNA methylation (GO:0044030);; Biological Process: histone H3-K14 acetylation (GO:0044154);; Biological Process: RNA-directed DNA methylation (GO:0080188);; |
| Zm00001d007745 | Molecular Function: protein serine/threonine kinase activity (GO:0004674);; Molecular Function: calcium ion binding (GO:0005509);; Molecular Function: ATP binding (GO:0005524);; Cellular Component: cell (GO:0005623);; Biological Process: cell surface receptor signaling pathway (GO:0007166);; Biological Process: oligosaccharide metabolic process (GO:0009311);; Biological Process: response to salicylic acid (GO:0009751);; Biological Process: unidimensional cell growth (GO:0009826);; Biological Process: cellular water homeostasis (GO:0009992);; Cellular Component: integral component of membrane (GO:0016021);; Molecular Function: polysaccharide binding (GO:0030247);; |
| MSTRG.28112 | -- |

| | |
|---|---|
| Zm00001d015759 | Molecular Function: DNA-binding transcription factor activity (GO:0003700);; Cellular Component: nucleus (GO:0005634);; Biological Process: transcription, DNA-templated (GO:0006351);; Biological Process: response to water deprivation (GO:0009414);; Biological Process: ethylene-activated signaling pathway (GO:0009873);; Biological Process: cutin biosynthetic process (GO:0010143);; Biological Process: wax metabolic process (GO:0010166);; Molecular Function: sequence-specific DNA binding (GO:0043565);; |
| MSTRG.26409 | Molecular Function: DNA binding (GO:0003677);; Molecular Function: glutathione transferase activity (GO:0004364);; Cellular Component: nucleus (GO:0005634);; Cellular Component: cytoplasm (GO:0005737);; Biological Process: toxin catabolic process (GO:0009407);; Biological Process: response to toxic substance (GO:0009636);; |
| Zm00001d049000 | Molecular Function: DNA binding (GO:0003677);; Cellular Component: nucleus (GO:0005634);; Biological Process: transcription, DNA-templated (GO:0006351);; Biological Process: regulation of transcription, DNA-templated (GO:0006355);; Molecular Function: metal ion binding (GO:0046872);; |
| Zm00001d045931 | -- |
| Zm00001d004209 | -- |

**Table S7.** Go annotation of 15 candidate genes in cQMES4.

| Gene ID | GO_annotation |
|---|---|
| Zm00001d051554 | Molecular Function: iron ion binding (GO:0005506);; Cellular Component: endoplasmic reticulum membrane (GO:0005789);; Biological Process: multicellular organism development (GO:0007275);; Biological Process: abscisic acid metabolic process (GO:0009687);; Biological Process: brassinosteroid homeostasis (GO:0010268);; Molecular Function: (+)-abscisic acid 8'-hydroxylase activity (GO:0010295);; Cellular Component: integral component of membrane (GO:0016021);; Biological Process: sterol metabolic process (GO:0016125);; Biological Process: brassinosteroid biosynthetic process (GO:0016132);; Molecular Function: oxidoreductase activity, acting on paired donors, with incorporation or reduction of molecular oxygen, NAD(P)H as one donor, and incorporation of one atom of oxygen (GO:0016709);; Molecular Function: heme binding (GO:0020037);; Biological Process: abscisic acid catabolic process (GO:0046345);; |
| Zm00001d051577 | Molecular Function: DNA binding (GO:0003677);; Molecular Function: DNA-binding transcription factor activity (GO:0003700);; Cellular Component: nucleus (GO:0005634);; Biological Process: transcription, DNA-templated (GO:0006351);; Biological Process: regulation of transcription, DNA-templated (GO:0006355);; Biological Process: procambium histogenesis (GO:0010067);; Biological Process: phloem or xylem histogenesis (GO:0010087);; Biological Process: positive regulation of transcription, DNA-templated (GO:0045893);; Molecular Function: metal ion binding (GO:0046872);; |
| Zm00001d051569 | Molecular Function: DNA binding (GO:0003677);; Molecular Function: DNA-binding transcription factor activity (GO:0003700);; Cellular Component: nucleus (GO:0005634);; Biological Process: transcription, DNA-templated (GO:0006351);; Biological Process: response to blue light (GO:0009637);; Biological Process: flower development (GO:0009908);; Biological Process: positive regulation of flower development (GO:0009911);; |
| Zm00001d051687 | Molecular Function: Rho GDP-dissociation inhibitor activity (GO:0005094);; Molecular Function: GTPase activator activity (GO:0005096);; Cellular Component: cytoplasm (GO:0005737);; Biological Process: cell tip growth (GO:0009932);; Biological Process: root epidermal cell differentiation (GO:0010053);; |
| Zm00001d051792 | Cellular Component: chromatin (GO:0000785);; Molecular Function: chromatin binding (GO:0003682);; Molecular Function: guanyl-nucleotide exchange factor activity (GO:0005085);; Cellular Component: nucleus (GO:0005634);; Cellular Component: cytosol (GO:0005829);; Biological Process: response to UV (GO:0009411);; Biological Process: entrainment of circadian clock (GO:0009649);; Molecular Function: photoreceptor activity (GO:0009881);; Biological Process: response to UV-B (GO:0010224);; Biological Process: protein-chromophore linkage (GO:0018298);; Molecular Function: protein homodimerization activity (GO:0042803);; |
| Zm00001d051787 | Cellular Component: endoplasmic reticulum (GO:0005783);; Biological Process: fatty acid biosynthetic process (GO:0006633);; Biological Process: response to cold (GO:0009409);; Biological Process: response to light stimulus (GO:0009416);; Molecular Function: fatty acid elongase activity (GO:0009922);; Cellular Component: membrane (GO:0016020);; Cellular Component: integral component of membrane (GO:0016021);; Biological Process: pollen tube development (GO:0048868);; Molecular Function: 3-oxo-arachidoyl-CoA synthase activity (GO:0102336);; Molecular Function: 3-oxo-cerotoyl-CoA synthase activity (GO:0102337);; Molecular Function: 3-oxo-lignoceronyl-CoA synthase activity (GO:0102338);; |
| Zm00001d051800 | Cellular Component: endoplasmic reticulum (GO:0005783);; Cellular Component: lipid droplet (GO:0005811);; Cellular Component: organelle membrane (GO:0031090);; Molecular Function: metal ion binding (GO:0046872);; Molecular Function: plant seed peroxidase activity (GO:1990137);; |
| Zm00001d051802 | Biological Process: auxin-activated signaling pathway (GO:0009734);; Biological Process: regulation of growth (GO:0040008);; |
| Zm00001d051829 | Molecular Function: ubiquitin-protein transferase activity (GO:0004842);; Cellular Component: nucleus (GO:0005634);; Cellular Component: cytoplasm (GO:0005737);; Cellular Component: plasma membrane (GO:0005886);; Biological Process: regulation of abscisic acid biosynthetic process (GO:0010115);; Biological Process: leaf senescence (GO:0010150);; Biological Process: regulation of chlorophyll catabolic process (GO:0010271);; Biological Process: regulation of chlorophyll biosynthetic process (GO:0010380);; Molecular Function: transmembrane receptor protein serine/threonine kinase binding (GO:0070696);; Biological Process: negative regulation of abscisic acid biosynthetic process (GO:0090359);; |
| Zm00001d051837 | Biological Process: sucrose metabolic process (GO:0005985);; Molecular Function: sucrose synthase activity (GO:0016157);; |
| Zm00001d051847 | Molecular Function: calmodulin binding (GO:0005516);; Cellular Component: vacuole (GO:0005773);; Cellular Component: vacuolar membrane (GO:0005774);; Cellular Component: endoplasmic reticulum (GO:0005783);; Cellular Component: plasma membrane (GO:0005886);; Biological Process: lignin metabolic process (GO:0009808);; Biological Process: unidimensional cell growth (GO:0009826);; Biological Process: plant-type secondary cell wall biogenesis (GO:0009834);; Cellular Component: membrane (GO:0016020);; Cellular Component: integral component of membrane (GO:0016021);; Biological Process: brassinosteroid biosynthetic process (GO:0016132);; Molecular Function: oxidoreductase activity, acting on CH-OH group of donors (GO:0016614);; Molecular Function: delta24-sterol reductase activity (GO:0050614);; Molecular Function: flavin adenine dinucleotide binding (GO:0050660);; |
| Zm00001d051520 | Molecular Function: DNA binding (GO:0003677);; Cellular Component: nucleus (GO:0005634);; Biological Process: transcription, |

| | |
|---|---|
| | DNA-templated (GO:0006351);; Biological Process: regulation of transcription, DNA-templated (GO:0006355);; |
| Zm00001d051529 | Molecular Function: ATP binding (GO:0005524);; Biological Process: phenylpropanoid metabolic process (GO:0009698);; Biological Process: pollen exine formation (GO:0010584);; Molecular Function: 4-coumarate-CoA ligase activity (GO:0016207);; |
| Zm00001d051925 | Molecular Function: protein serine/threonine kinase activity (GO:0004674);; Molecular Function: transmembrane receptor protein serine/threonine kinase activity (GO:0004675);; Molecular Function: ATP binding (GO:0005524);; Cellular Component: plasma membrane (GO:0005886);; Biological Process: cell surface receptor signaling pathway (GO:0007166);; Biological Process: protoderm histogenesis (GO:0010068);; Cellular Component: integral component of membrane (GO:0016021);; Molecular Function: kinase activity (GO:0016301);; Biological Process: cell differentiation (GO:0030154);; Biological Process: cuticle development (GO:0042335);; Biological Process: protein autophosphorylation (GO:0046777);; Biological Process: plant organ formation (GO:1905393);; |
| Zm00001d051928 | Molecular Function: structural constituent of nuclear pore (GO:0005487);; Molecular Function: phospholipid binding (GO:0005543);; Cellular Component: nuclear envelope (GO:0005635);; Cellular Component: cytosol (GO:0005829);; Biological Process: RNA export from nucleus (GO:0006405);; Biological Process: protein import into nucleus (GO:0006606);; Biological Process: negative regulation of auxin mediated signaling pathway (GO:0010930);; Molecular Function: structural constituent of nuclear pore (GO:0017056);; Cellular Component: nuclear periphery (GO:0034399);; Cellular Component: nuclear pore central transport channel (GO:0044613);; Biological Process: mRNA transport (GO:0051028);; Biological Process: ribonucleoprotein complex export from nucleus (GO:0071426);; |

**Table S8.** Accession numbers to NCBI database.

| | |
|---|---|
| Accession | PRJNA822510 |

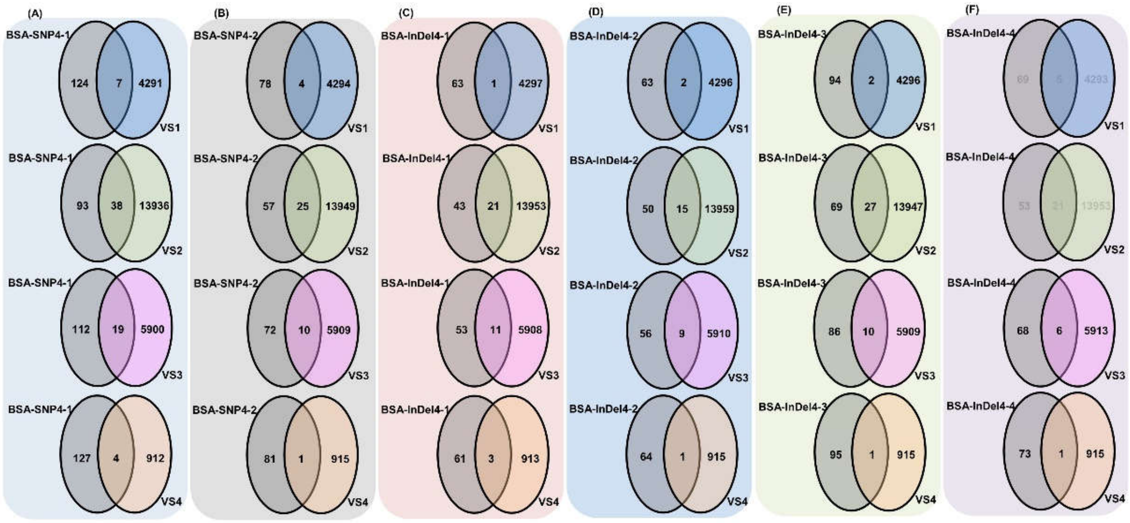


**Figure S1.** Veen diagram of genes between BSA-SNP4-1 (A), BSA-SNP4-2 (B), BSA-InDel4-1 (C), BSA-InDel4-2 (D), BSA-InDel4-3 (E), and BSA-InDel4-4 (F) region by BSA strategy and four comparisons (VS1 [W64A(20 cm)_VS_K12(20 cm)], VS2 [W64A(3 cm)_VS_K12(3 cm)], VS3 [W64A(20 cm)_VS_W64A(3 cm)], and VS4 [K12(20 cm)_VS_K12(3 cm)]) via RNA-sequencing, respectively.
